# Acquired Angioedema Post COVID-19 Infection: Can SARS-Cov-2 Induce Angioedema?

**DOI:** 10.7759/cureus.70951

**Published:** 2024-10-06

**Authors:** Misleydi Rios Rodriguez, Jake Singh, Kindness Nwakudu, Shannon Feely, Anila Khaliq, David Manna

**Affiliations:** 1 Family Medicine, Touro College of Osteopathic Medicine, Middletown, USA; 2 Family Medicine, Garnet Health Medical Center, Middletown, USA; 3 Basic Biomedical Science, Touro College of Osteopathic Medicine, Middletown, USA

**Keywords:** angioedema, covid-19, hereditary angioedema, non-respiratory symptoms post covid, post-covid

## Abstract

Ongoing research continues to uncover the long-term effects of post-COVID-19 illnesses. Patients with hereditary angioedema (HAE) are believed to have an elevated risk of contracting COVID-19 caused by SARS-CoV-2, potentially leading to increased frequency or more severe symptoms. This speculation is based on the understanding that COVID-19 enters cells through the angiotensin-converting enzyme 2 (ACE2) receptor. This report presents the case of a 37-year-old man with no significant medical history who presented to the clinic with what was initially thought to be an allergic reaction. He had experienced swelling in his lips, both upper extremities, and widespread urticaria, along with mild associated abdominal pain. While there is currently no conclusive evidence linking COVID-19 to the onset of HAE, a significant number of cases suggest a potential connection between post-COVID-19 angioedema and long-term angioedema. Subsequently, the patient experienced a second episode of angioedema flare-up aggravated by an upper respiratory infection.

## Introduction

Discussions surrounding the interplay between hereditary angioedema (HAE) and infection by COVID-19 have gained traction. HAE is a rare autosomal dominant disorder characterized by bradykinin-mediated, nonpruritic swelling of the skin, upper airway, and gastrointestinal tract [1-3]. The pathophysiology of HAE involves the deficiency or dysfunction of the C1 esterase inhibitor (C1-INH), resulting in increased levels of bradykinin, a potent vasodilator [3-6]. 

Studies suggested that COVID-19 triggered angioedema attacks, potentially exacerbating symptoms in patients with HAE [1,3]. Hausburg et al. theorized that COVID-19 viral proteins might bind to C1-INH, reducing their activity [4]. Loss of C1-INH activity leads to overactivation of the plasma kallikrein-kinin system, increasing bradykinin levels and subsequently increasing vascular permeability and inflammation by generating prostaglandins and nitric oxide [7-9]. Additionally, COVID-19 enters the cells through the angiotensin-converting enzyme 2 (ACE2) receptor, leading to rising levels of bradykinin and decreasing ACE levels. These findings raise concerns about the severity of COVID-19 in patients with HAE [3,9]. Therefore, it is plausible that COVID-19 could exacerbate or induce HAE-like episodes in patients without a prior history of related symptoms.

## Case presentation

A 37-year-old man with no significant medical history, except for a prior COVID-19 infection and one angioedema episode a year ago, came in for a follow-up appointment after visiting the emergency department. He had visited the ED about four weeks ago for what was initially believed to be an allergic reaction. At the time of the ED visit, he experienced swelling of the lips and both upper arms, widespread hives, and mild abdominal pain. Laboratory tests at the emergency department showed no abnormalities, and he was treated symptomatically and discharged home in stable conditions after a six-hour observation period. This episode of angioedema occurred five to six days after the patient experienced a "cold," which lasted for approximately five days with symptoms of rhinorrhea, dry cough, mild subjective fever, and body aches. 

Approximately one year ago, the patient had his first episode of angioedema, which occurred five days after he was diagnosed with COVID-19 at home and confirmed at the ED. He reported experiencing the same symptoms of swelling of the lips and both upper arms, widespread hives, and mild abdominal pain. This was the first time the patient experienced symptoms mimicking allergic reactions. He denied any prior similar episodes, as well as a history of asthma or any atypia symptoms. He reported no family history of angioedema, asthma, or atypia. Laboratory tests during this past ED visit showed no abnormalities, and he was treated for symptom relief and discharged home with close follow-up. 

Unlike the first episode, which occurred one year ago, the most recent second episode of angioedema was not confirmed to be following COVID-19, leading to the consideration of a newly developed angioedema condition or a non-specific viral trigger for symptoms resembling hereditary angioedema.

## Discussion

COVID-19 has been associated with various dermatological conditions, including vesicles, lesions, petechiae, exanthems, and urticaria; however, angioedema has been infrequently reported [5,10,11]. Several documented cases of angioedema following COVID-19 exposure highlight the need for further research on this connection. For example, a 62-year-old male experienced exacerbated angioedema of the eyes, lips, and cheeks 12 days after COVID-19 exposure without prior history [11]. Similarly, a 30-year-old woman had lip and periocular swelling 11 days post COVID-19 infection, with quick recovery following antihistamine treatment [12], as observed in our patient.

**Figure 1:**
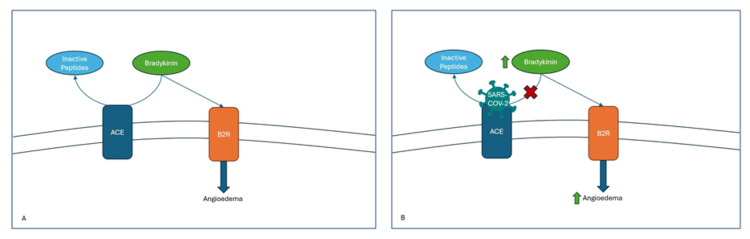
(A) Normal pathway of bradykinin degradation by ACE and its interaction with the B2R receptor leading to angioedema; (B) Interaction of SARS-CoV-2 with ACE leading to increased bradykinin levels and heightened risk of angioedema. ACE: angiotensin-converting enzyme; B2R: type 2 bradykinin receptor

The precise mechanism by which COVID-19 may induce angioedema remains unclear, but potential pathways include the interplay between ACE and bradykinin (Figure 1). ACE inhibitor-induced angioedema is believed to be mediated by increased bradykinin levels due to impaired breakdown by ACE [7,13,14]. The primary metabolizer of bradykinin is ACE1, and one of the breakdown products, des-Arg9-bradykinin, is further metabolized by ACE2 [15-17]. Impairment in this metabolic pathway can lead to an accumulation of bradykinin, resulting in increased vascular permeability and angioedema (Figure 1B). This mechanism may explain the development of HAE-like symptoms in COVID-19 patients.

The authors recommend further exploration of COVID-19's potential to induce HAE-like disease. Increasing awareness of symptoms and management strategies is crucial to prevent complications. The timeline and presentation suggest that acquired HAE-like disease can emerge in patients without a prior history.

## Conclusions

Despite the lack of conclusive evidence, there have been numerous cases suggesting a potential link between post-COVID angioedema and long-term angioedema. This intriguing case study sheds light on the possibility of COVID-induced angioedema as a plausible explanation for the patient's presentation. The patient experienced two episodes of angioedema-like flares, one year apart, both following upper respiratory infections. While the first episode was confirmed as a COVID-19 infection, the second was not. It remains unclear if this exacerbation was due to another acute COVID-19 infection or a newly acquired angioedema condition that may flare with various viral infections unrelated to COVID-19. The fascinating mechanism of SARS-CoV-2 protein S binding to the ACE2 receptor, facilitating viral entry into cells, may be connected to bradykinin degradation products, providing valuable insight into cases like this one.
